# Clark on Puerperal Peritonitis

**Published:** 1855-04

**Authors:** 


*Clark on Puerperal Peritonitis.*—Prof. Clark communicates to the new American edition of Ramsbotham's System of Obstetrics, a paper detailing his views of the opium treatment. We abstract a portion of his conclusion:

1st. When a prominent element in "puerperal fever" is peritonitis, the treatment with large doses of opium is more successful than any other that has yet been proposed.

2d. To be successful, this treatment must be commenced early, and the patient must be brought under its influence as rapidly as the susceptibility of the system can be ascertained by trial.

3d. The quantity of opium required to produce a safe but desirable degree of narcotism, varies greatly in different cases; so that it is necessary to begin with doses that cannot do mischief, and increase every two hours till the influence of the opiate is sufficiently decided.

4th. Every dose, during at least the whole tentative period, should be administered by the physician himself, or by some person on whose knowledge of the effects of opium and whose watchfulness and discretion he can rely. Some young physicians are too bold, and endanger the life of the patient; others are too timid, and do not control the disease.

5th. The opium treatment alone will not cure "puerperal fever," when its leading element is purulent metritis, though there is reason to believe that it will control, and even prevent the peritonitis which generally accompanies it. This conclusion has been confirmed by recent observations.

6th. The tolerance of opium in some cases of puerperal peritonitis almost surpasses belief. Yet in private practice I have not found more than half or two-thirds of a grain of sulph. morph., every two hours, necessary, and have generally begun with less, except for the first dose.

7th. The influence of the opium should be kept up till the pain and tenderness subside, the tympanitis diminishes in some degree, and the pulse falls below 100—then, with the concurrence of other symptoms, it should be gradually diminished, and it length discontinued.

Alluding to the enormous doses of opium endured by some of the cases he details, Prof. Clark says that subsequent experience has proved that such treatment is unnecessary, and that all the beneficial effect of the drug may be obtained by more moderate doses. We do not understand him, however, to recede from the position that the patient should be narcotized, or at least carried to the verge of narcotism.

He speaks thus rationally and moderately of the success of the treatment:

"The results of the opium treatment in the hands of my professional friends in this city have not been uniformly successful. This was to have been expected. When the path to success is so narrow, and so little trodden, though beset with dangers on both sides, it is unavoidable that many will lose it. But I believe I am authorized in saying, that those who have seen most of this mode of medication, are most attached to it. It is not to be expected that in a disease so dangerous as the one under consideration, any plan can be uniformly successful, even with advantages of accurate diagnosis and early treatment; but, when it is remembered that the diagnosis between purulent metritis and puerperal peritonitis is not always easy,—and that this medication is successful in proportion to its early adoption,—we may probably find reason for its failure in other hands, as well as in my own.

"By way of illustrating the vigilance and discretion which must be exercised in the administration of each successive dose of the opiate in this mode of treatment, I will add, that it could never have been fairly tested by me without the zealous, intelligent, and untiring assistance of the house-physicians of the hospital. They visited the patients every hour by night as well as by day, and every dose of the medicine, from the first case to the last, was given by them, and proportioned to the hourly exigencies." H.

